# Scanning Strategies Do Not Modulate Face Identification: Eye-Tracking and Near-Infrared Spectroscopy Study

**DOI:** 10.1371/journal.pone.0011050

**Published:** 2010-06-10

**Authors:** Yosuke Kita, Atsuko Gunji, Kotoe Sakihara, Masumi Inagaki, Makiko Kaga, Eiji Nakagawa, Toru Hosokawa

**Affiliations:** 1 Graduate School of Education, Tohoku University, Sendai, Japan; 2 Research Fellow of the Japan Society for the Promotion of Science, Tokyo, Japan; 3 Department of Developmental Disorders, National Institute of Mental Health, National Center of Neurology and Psychiatry (NCNP), Tokyo, Japan; 4 Department of Child Neurology, National Center Hospital of Neurology and Psychiatry, National Center of Neurology and Psychiatry (NCNP), Tokyo, Japan; University of Granada, Spain

## Abstract

**Background:**

During face identification in humans, facial information is sampled (seeing) and handled (processing) in ways that are influenced by the kind of facial image type, such as a self-image or an image of another face. However, the relationship between seeing and information processing is seldom considered. In this study, we aimed to reveal this relationship using simultaneous eye-tracking measurements and near-infrared spectroscopy (NIRS) in face identification tasks.

**Methodology/Principal Findings:**

22 healthy adult subjects (8 males and 14 females) were shown facial morphing movies in which an initial facial image gradually changed into another facial image (that is, the subject's own face was changed to a familiar face). The fixation patterns on facial features were recorded, along with changes in oxyhemoglobin (oxyHb) levels in the frontal lobe, while the subjects identified several faces. In the self-face condition (self-face as the initial image), hemodynamic activity around the right inferior frontal gyrus (IFG) was significantly greater than in the familiar-face condition. On the other hand, the scanning strategy was similar in almost all conditions with more fixations on the eyes and nose than on other areas. Fixation time on the eye area did not correlate with changes in oxyHb levels, and none of the scanning strategy indices could estimate the hemodynamic changes.

**Conclusions/Significance:**

We conclude that hemodynamic activity, i.e., the means of processing facial information, is not always modulated by the face-scanning strategy, i.e., the way of seeing, and that the right IFG plays important roles in both self-other facial discrimination and self-evaluation.

## Introduction

Humans can quickly identify faces within a glimpse. The cognitive process of face identification contributes to the social cognition and social skills acquisition related to self-recognition, and facilitates communication with others. Previous studies have implicated the role of structural encoding and semantic processing in face identification. Bruce [1] reported that familiar faces are recognized more quickly than unfamiliar faces. Familiarity with specific facial images is also an aspect of social cognition, which is acquired within 45 h [2] or at two days [3] after birth in human infants, and the capacity for self-face (one's own face) recognition subsequently increases until two years of age. For example, toddlers can recognize a facial image in a mirror as their own face [4]. A self-face is identified faster than unfamiliar faces [5] and also has a strong tendency to gain the participant's attention; participants look at their own face for a longer time than that of an unfamiliar face [6], or find it harder to ignore than a familiar face [7]. These behavioral findings suggest that the face identification is modulated by social context, that is, self–other distinction and familiarity.

To explain the neurophysiological modulation of face identification, two approaches have been explored. One of them is the measurement of eye movements. Previous studies reported several face-scanning strategies; for example, subjects were more likely to fixate on internal facial features of a familiar face than on those of an unfamiliar face [8], [9]. In particular, more time was spent fixating the eye area of familiar faces than of unfamiliar faces [10]. Eyes are an important feature in face recognition [11] and thus, the absence of the eye or eyebrow areas makes recognizing familiar faces difficult [12]. Note that these studies implicated local scanning strategies for familiar faces, which suggests characteristic face identification to extract semantic information.

Another approach is noninvasive neuroimaging technique which identifies the cortical and subcortical pathways used during face identification. Recently, many studies showed the association of several brain regions with semantic encoding of faces [13], [14]. For instance, in self-face recognition, activation is often seen in the fusiform gyrus [15], precuneus [16], and frontotemporal regions [17]. This activation indicates parallel and/or sequential interaction among the brain regions, which in turn may play a crucial role in mediating face identity node and higher cognitive processes such as attention, memory retrieval, and discrimination required by task demands. Indeed, discrimination tasks involving self and other faces markedly activate the inferior frontal gyrus (IFG) in the right hemisphere but not in the left hemisphere [18], [19].

Regarding relationships between information sampling as a peripheral function and information processing as a brain function, Dalton et al. [20] reported interesting findings. In their study, typical individuals and autistic patients performed a face discrimination task while information sampling and processing were measured with an eye-tracking method, and by functional magnetic resonance imaging (fMRI). Autistic patients faced difficulties in social communication, as typified by fewer eye contacts or excessive gazing at specific facial features. Those results showed positive correlations between the duration of fixation on eyes areas and activity either in the right amygdala or the right anterior fusiform gyrus in the autistic group. However, typical individuals do not show significant correlations between fixation and activity because of a ceiling effect for eye-fixation. Thus, Morris and colleagues [21], [22] explored the subjects' scanning strategies to a facial image after instructing them to fixate an area in a regular scanning strategy for a fixed duration, in order to explore the connections of scanning strategy and brain activities. They found that typical scanning strategy (mainly fixation on the eye and mouth areas) intensified activity in the fusiform gyrus compared with atypical strategy, such as fixation on the cheek, chin, or forehead instead of the eyes or mouth.

These three findings indicated the existence of a functional relationship between scanning strategy and brain activity in specific areas. Thus, a long duration of gaze on the eye area for familiar faces [8], [9], [10] could increase activity in the amygdala and fusiform gyrus. It is important to determine whether familiarity enhances activation in the prefrontal cortex, which is thought to be linked to self-recognition [18], [19]. Here, we studied interactive involvement in facial informational sampling and processing, focusing on the prefrontal cortex including the IFG in face identification tasks by the means of simultaneous eye-tracking and near-infrared spectroscopy (NIRS). NIRS is a noninvasive neuroimaging technique which can measure hemodynamic activity in the cortex, and the oxygen extraction fraction is reported to parallel the blood-oxygen-level-dependent (BOLD) signal measured by fMRI [23]. This technique enables us to explore brain activities especially in prefrontal cortex area [24]–[26]. In addition, with NIRS it is not necessary to fix the subjects' body position accurately, as is required for fMRI or positron emission tomography [27]. Hence, we were able to perform simultaneous measurements in a natural setting, not in overly-stressful setting for subjects.

## Materials and Methods

### Subjects

Twenty-two healthy adults acted as paid volunteers (8 males and 14 females, aged 22.9±2.5 years) participated in this study. All subjects were right-handed, had normal or corrected to normal vision, and had no history of neurological or psychiatric disorders. All subjects gave written informed consent before the experiment, which was approved by the Ethics Committee of National Center of Neurology and Psychiatry (NCNP), Japan.

### Stimuli

We used morphing movies as stimuli in the face identification tasks, in which an initial image changed dynamically to a target image [28]. In order to make face-morphing movies, three facial images were prepared for each subject: a self-face, a familiar face, and an unfamiliar face. These were prepared from photographs taken with a digital camera (resolution 72 dots per inch). The self-face image was a mirror image of the subject's own face. Each of the familiar face images was of a friend or coworker of a subject, whom the subject saw several times per week. The image was gender- and age-matched for each subject. The unfamiliar face image was an image of an average face based on people that the subject had never seen, which were created by Software for Facial Image Processing System for Human-like “Kansei” Agent (Information-technology Promotion Agency, Japan) and an extension tool (Harashima-Naemura Laboratory, University of Tokyo, Tokyo, Japan). Two types of unfamiliar faces were prepared according to the subject's gender: male (from 12 young men, aged 20–23 years) and female (from 11 young women, aged 20–26 years). All facial images were changed to monochromatic photographs, devoid of apparent features such as glasses and a moustache, and were of oval in shape showing main features, such as both eyes, the nose, and the mouth but not the hair or ears. We equalized the average luminance of all facial images using a commercially available software such as Adobe Photoshop CS (Adobe Systems Inc., CA, USA), and the luminance was kept constant while the movies were shown to the participants. Before the experiment, we confirmed person identification to the stimuli using static facial images in each subject.

**Figure 1:**
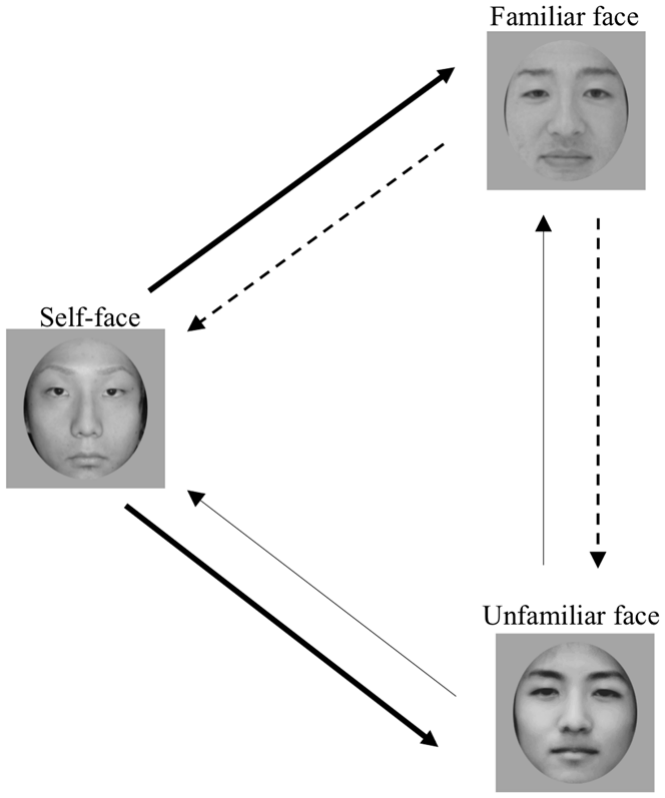
Conditions of the face identification task. Directions of each arrow show the course of the morphing movies (starting from the initial image to target image): 1) Self-face condition: from self-face to familiar or unfamiliar face (thick arrows). 2) Familiar-face condition: from familiar face to self or unfamiliar face (dashed arrows). 3) Unfamiliar-face condition: from unfamiliar face to self or familiar face (thin arrows). Both subjects of self- and familiar facial images have given written informed consents (as outlined in the PLoS consent form) to publication of their picture. Unfamiliar facial image was artificially created (nonexistent person).

Each morphing movie was created from one pair of the three facial images using WinMorph 3.01 (debug mode: http://www.debugmode.com). A total of six movie patterns were thus created for each subject, as follows: from self to familiar or unfamiliar; from familiar to self or unfamiliar; and from unfamiliar to self or familiar (Fig. 1). We used the eyes, eyebrows, nose, mouth, and an outline of the facial images as reference items for adjustment to match the principal components of two different facial images. A total of 200 morphed frames were generated for each pair of images; each successive frame represented a 0.5% change from one image to the next.

The movie stimuli were presented in the center of a gray background on a 15-inch PC LCD monitor (Dell Inc.; display resolution was 1280 pixels (width)×1024 pixels (height), refresh rate was 60 Hz) using Experiment Builder version 1.3.40 (SR Research Ltd., Mississauga, Canada). Size of the movie image was 102 mm (width)×122 mm (length) (visual angle 9.7°×11.6°). The distance between the PC monitor and the eyes of the subject was 600 mm. Movie frames were displayed at 10 frames per second and the display duration of each movie was 20 s.

### Task

**Figure 2:**
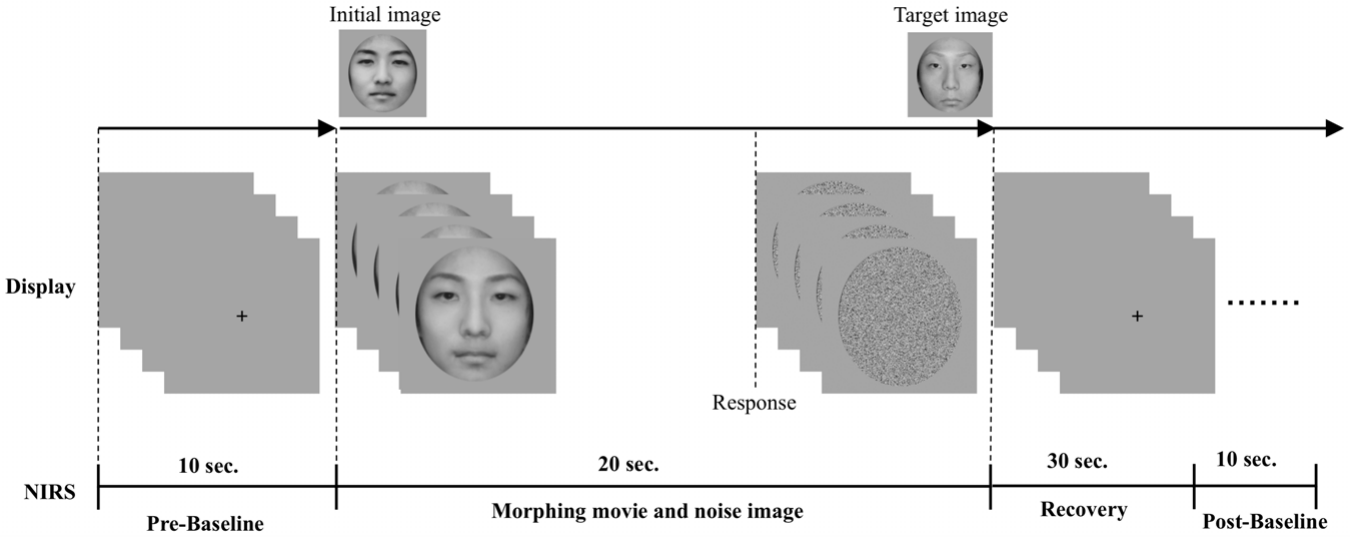
Time course of one trial. Subject saw fixation cross for 10 s and the morphing movie started successively. The subject pressed the key when he/she thought that the initial image had changed into the target image and then static noise image appeared for the rest of 20 s. After the movie and noise image, the subject looked at the PC monitor for almost 40 s. We set two baseline data, pre-baseline and post-baseline, to correct the baseline of raw NIRS data with linear fitting.

Each subject was required to see a black fixation cross (10 mm×10 mm) appeared in the center of the screen for 10 s (Fig. 2). Then, the subject was instructed to watch the morphing movie and to press a key button with the right index finger when he or she thought that the initial facial image had changed into the target image. When the subject pressed the key button, a static noise image appeared immediately instead of the movie. Twenty seconds after the initial image was shown, we instructed the subject to continue looking at the PC monitor for a time period longer than 40 s. Thus, one trial was almost for 70 s and next trial was started successively.

Tasks were divided into three conditions, as follows: 1) self-face changing to familiar or unfamiliar face; 2) familiar face changing to self or unfamiliar face; and 3) unfamiliar face changing to self or familiar face (Fig. 1). Each morphing movie was presented at least three times, so that each subject viewed more than six trials (two morphing movies×three presentations) in each condition. The conditions and trials were shown in a random order to the subjects.

### Recordings

#### Eye-tracking

Real-time eye movements were recorded using EyeLink Remote (SR Research Ltd.) with a monocular sampling rate of 500 Hz. A small target sticker was affixed to the forehead of each subject above the eye being recorded, which allowed the head position to be tracked even when the pupil image was lost. Before initiating the experiment, we adjusted the calibration of the camera to monitor the pupil of the subject's eye. Then, we calibrated eye fixation manually using a nine-point fixation procedure, in which a small black dot was appeared in random order at a corner on the PC monitor or at a midpoint between the corners. In the calibration procedure, the subject was instructed to fixate the dot for more than 1000 ms, and we checked eye-tracking in the validation procedure in the same way as for the calibration procedure. These procedures were repeated until an optimal recording situation was confirmed.

#### Near-infrared spectroscopy (NIRS)

**Figure 3:**
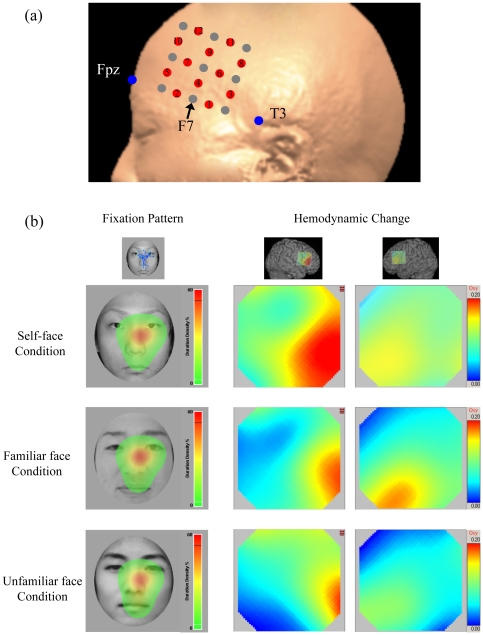
Location of NIRS probes and channels (a) and Fixation pattern maps and topographies for changes in oxyHb levels in one representative subject (b). (a) Five emission and four detector probes (gray dots) were arranged in a 3×3 square lattice and the three lowest probes were aligned between Fp1/Fp2 and T3/T4 (blue dots). The mid probe of the three was placed around F7/F8 (international 10/20 system). We obtained cortical responses from a total of 12 channels (red dots) in each hemisphere. Numbers in red dots show channel numbers, i.e. 1 means Ch. 1. (b) Hemodynamic activities in the right hemisphere, especially the areas corresponding with inferior frontal gyrus, were higher in the self-face condition compared with other conditions, but fixation patterns were similar among all conditions. Each line presents a condition: the upper line shows the self-face condition, the middle line shows the familiar face condition, and the lowest line shows the unfamiliar face condition. The left column shows fixation maps and the other two columns show hemodynamic changes, the middle column for the right hemisphere and right column for the left hemisphere. A two-dimensional Gaussian distribution was applied to each of the fixation maps created. The center of this Gaussian distribution is the fixation location and its width is set to 1° of visual angle: the height of the Gaussian is weighted by the proportion of dwell time on each area. Both subjects of self- and familiar facial images have given written informed consents (as outlined in the PLoS consent form) to publication of their picture. Unfamiliar facial image was artificially created (nonexistent person).

Changes in oxyhemoglobin (oxyHb) levels were recorded using the ETG-4000 (Hitachi Medical Corp., Tokyo, Japan) with 24 channels. Present system used two wavelengths, approximately 695 and 830 nm, of near-infrared light whose absorption was recorded to estimate oxyHb levels. The temporal resolution was set at 100 ms. The emission probes were located 3.0 cm from the detector probes. This system could measure changes in oxyHb levels at a depth of 2–3 cm below the scalp [29]. Five emission and four detector probes were arranged in a 3×3 square lattice on each lateral forehead; thus, cortical responses were obtained from a total of 12 locations in each hemisphere (Fig. 3(a)). The three lowest probes were aligned between Fp1/Fp2 and T3/T4 in accordance with the international 10/20 system used in electroencephalography, and the mid-probe was placed around F7/F8.

### Analysis

Reaction time (RT) was analyzed in each subject as the behavioral variable using the EyeLink Data Viewer (SR Research Ltd.) and was measured as the duration from the onset of the morphing movie until the subject pressed the key. Eye-tracking and NIRS data were excluded from the analysis when the subject did not press the key.

Eye-tracking data during the task were analyzed for the average fixation time for each fixation point, the average distance at which the stimulus was viewed, and the fixation time and the number of fixations in the facial regions of interest (fROIs). A fixation was defined as the continuous period of at least 100 ms spent looking within 1° of visual angle, according to a previous study [30]. The distance was defined as the saccade amplitude between consecutive fixations, and the averaged distance was calculated for each consecutive fixation in every trial. fROIs were arranged near four areas: eyes (right eye and left eye), nose, mouth, and other (facial areas except in eyes, nose and mouth). fROIs of the eyes, nose, and mouth had the same area sizes, which enabled us to compare the fixation time and fixation counts of each fROI precisely.

For NIRS data, trials with artifacts caused by body movement and inappropriate probe settings were excluded before analysis. The baseline of raw NIRS data in each trial was corrected by linear fitting procedure based on the two baseline data: the mean across a 10-s-period just before the morphing movies, and the mean across a final 10-s period of 40 s after the noise image (Fig. 2). Then, we filtered the NIRS data by low-pass filter (0.5Hz) and moving averages with a 5-s time window to reject artifacts caused by minor movement of the subject. To determine oxyHb levels, changes in the self-face specific region of the lateral prefrontal cortex [18], [19], ROIs for NIRS data (nROIs) were arranged with two regions: 1) left inferior frontal gyrus (L-IFG: channels 1, 2, 4, and 5); 2) right inferior frontal gyrus (R-IFG: channels 13, 14, 15, and 16). Changes in oxyHb levels in each nROI were averaged in each condition.

Behavioral and eye-tracking data (only for the average fixation time and the average distance) were analyzed using one-way repeated measures analysis of variance (ANOVA) with conditions (self-face, familiar face, and unfamiliar face) as the independent variable, followed by post hoc analysis using Bonferroni adjustment. Eye-tracking data (for fixation time and counts in fROIs) were analyzed using two-way repeated measures ANOVA with conditions (self-face, familiar face, and unfamiliar face) and fROI (eyes, nose, and mouth) as independent variables in a similar way. For NIRS data, we subtracted changes in oxyHb levels in the self-face condition from those in the unfamiliar-face condition (Self minus Unfam, Self-Unfam) and the changes in the familiar-face condition from those in the unfamiliar-face conditions (Fam minus Unfam, Fam-Unfam) in order to remove the effects of face recognition itself in IFG. These changes in oxyHb levels in stimuli intervals were analyzed by two-way repeated measures ANOVA with condition (Self–Unfam and Fam–Unfam) and nROI (Left-IFG and Right-IFG) as independent variables, followed by post hoc analysis using Bonferroni adjustment. To explore the relation between fixation patterns and hemodynamic activities, we applied multiple linear regression analysis to changes in oxyHb levels in each nROI as dependent variables with ratios of fixation time to total fixation time in each fROI as independent variables, and also calculated Pearson's *r* value with NIRS data and fixation time in the eye area [11]. Data processing and statistical analyses were performed with Matlab 7.8 (The Mathworks Inc., MA, USA) and PASW version 18.0 (SPSS Japan Inc., Tokyo, Japan).

## Results

### Behavioral data

All subjects could identify the three facial images before the task, and they responded accurately to the morphing movie within 20 s in every condition. The RTs for self-face, familiar face and unfamiliar face were 14259.1±432.1 (mean±standard error of the mean (SEM)) ms, 13969.7±399.1 ms, and 14607.4±449.4 ms, respectively. There was no significant main effect of the condition (*F*(2,42) = 1.4, n.s.) (Table 1).

**Table 1:** Reaction time (RT), average fixation time for each fixation point, and average distance between consecutive fixations in each condition.

Condition	RT (ms)	Average fixation time/point (ms)	Average distance between fixations (°)
Self-face	14259±432	424.1±13.4	2.3±0.1
Familiar face	13970±399	421.1±13.8	2.2±0.1
Unfamiliar face	14607±449	423.7±21.3	2.2±0.1

Each value shows mean scores±standard error of the mean (SEM).

### Eye-tracking data

The average fixation times per fixation point were 424.1±13.4 ms for self-face, 421.1±13.8 ms for familiar face, and 423.7±21.3 ms for unfamiliar face. There was no significant main effect of the condition (*F*(2,42)<1.0, n.s.). The average distance per consecutive fixation point was 2.3±0.1° for self-face, 2.2±0.1° for familiar face, and 2.2±0.1° for unfamiliar face. There was also no significant main effect of the condition (*F*(2,42)<1.0, n.s.) (Table 1). Eye-tracking data for one individual are shown in Fig. 3(b).

**Figure 4:**
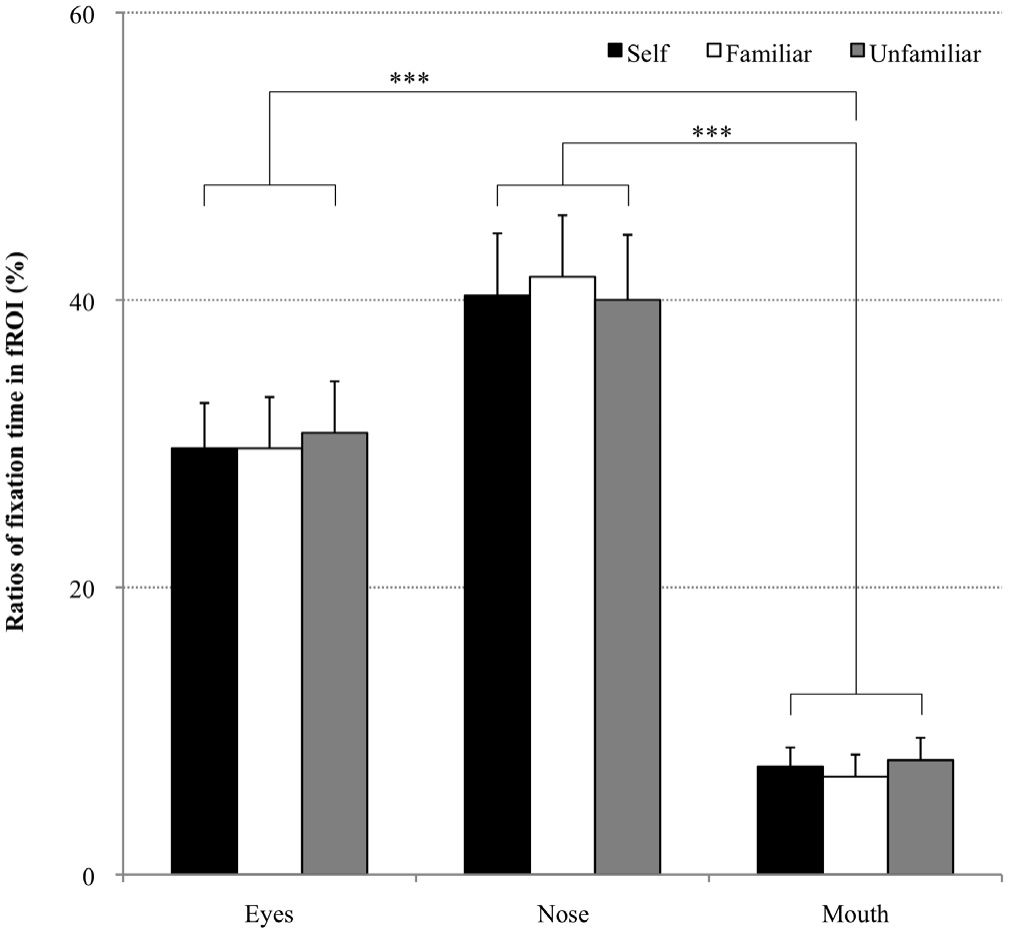
Ratios of fixation time in the facial region of interest (fROI) to total fixation time. Fixation time for the eyes and nose is significantly longer than for the mouth area (*p*<0.001), though the there is no significant difference between fixation time in each condition. *** *p*<0.001.

The ratios of fixation time in each fROI were as follows: self-face, 29.7±3.1% (eyes), 40.3±4.3% (nose), and 7.5±1.3% (mouth); familiar face, 29.7±3.6% (eyes), 41.6±4.3% (nose), and 6.8±1.5% (mouth); and unfamiliar face, 30.8±3.6% (eyes), 40.0±4.5% (nose), 8.0±1.6% (mouth). The main effect of fROI was significant (*F*(2,42) = 19.5, *p*<0.001) and post hoc analysis revealed that values for the mouth were significantly different from those for the eyes and nose (*p*<0.001). There was no significant main effect of the condition (*F*(2,42)<1.0, n.s.) and no interaction (*F*(4,84)<1.0, n.s.) (Fig. 4).

The ratios of fixation counts in each fROI to total fixation counts were as follows: self-face, 29.8±3.0% (eyes), 39.5±4.1% (nose), and 7.0±1.2% (mouth); familiar face, 29.7±3.4% (eyes), 40.8±4.1% (nose), and 6.4±1.4% (mouth); and unfamiliar face, 30.6±3.5% (eyes), 40.0±4.4% (nose), and 7.3±1.5% (mouth). The main effect of fROI was significant (*F*(2,42) = 21.2, *p*<0.001) and post hoc analysis revealed that values for the mouth were significantly different from those for the eyes and nose (*p*<0.001). There was no significant main effect of the condition (*F*(2,42)<1.0, n.s.) and no interaction (*F*(4,84)<1.0, n.s.).

### NIRS data

**Figure 5:**
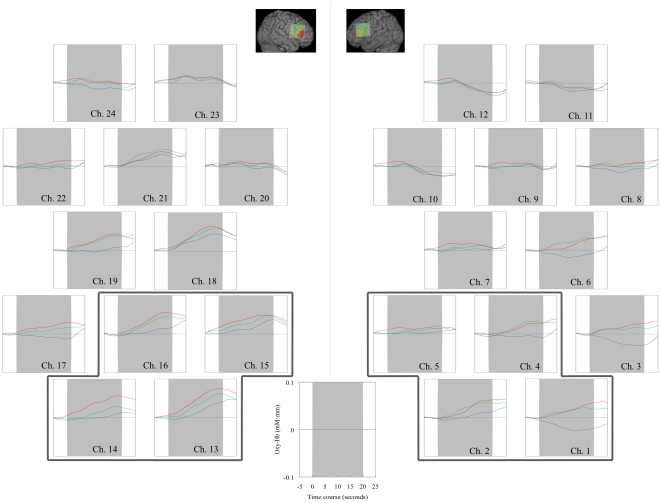
Grand average waveforms in three conditions. Changes in oxyHb levels in the area corresponding to the right inferior gyrus (right area surrounded by a bold line) were greater in the self-face condition (red wavelike lines) than in the familiar- (green wavelike lines) and unfamiliar- (blue wavelike lines) face conditions. Gray areas indicate the 20-s task period and areas enclosed by bold lines are ROIs for hemodynamic activities (nROI: right for R-IFG; left for L-IFG).

Representative patterns of hemodynamic activities in a male subject are shown in Fig. 3(b), and grand average waveforms for changes in oxyHb level of each condition are shown in Fig. 5.

**Figure 6:**
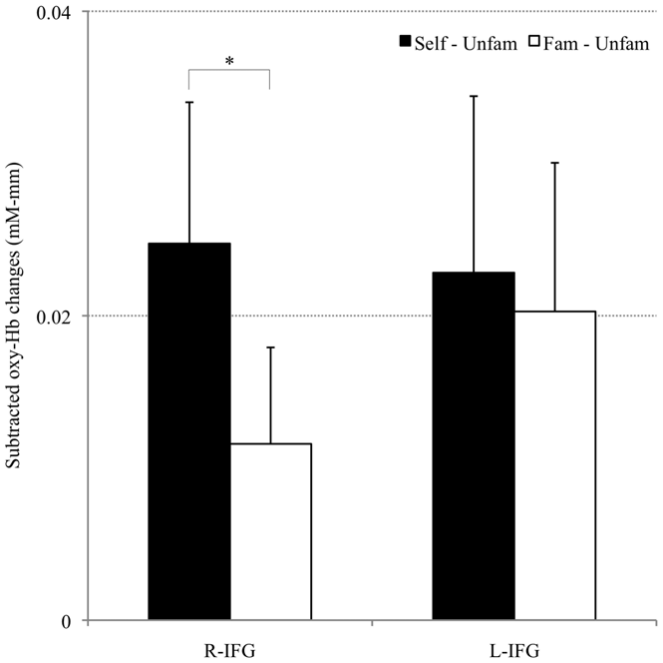
Subtracted changes in oxyHb levels in each nROI. Changes in oxyHb levels for the unfamiliar-face condition subtracted from the self-face condition (Self – Unfam) show significant increments compared with the unfamiliar-face condition subtracted from the familiar-face condition (Fam – Unfam) in the R-IFG but not in the L-IFG. (R-IFG: right for R; left for L-IFG). *p<0.05.

There was a significant interaction of nROI and condition (*F*(1,21) = 4.4, *p*<0.05) and post hoc analysis revealed that on the right side the subtracted changes for Self – Unfam (0.025±0.01 mM•mm) were higher than for Fam–Unfam (0.012±0.01 mM•mm) (*p*<0.05). The differences between outcomes of other comparisons were not significant. Main effects of neither condition (*F*(1,21) = 1.7, n.s.) nor nROI were significant (*F*(1,21)<1.0, n.s.) (Fig. 6). Moreover, no significant main effects of condition (*F*(1,21)<1.0, n.s.) or nROI (*F*(1, 21)<1.0, n.s.) were detected, and no interaction (*F*(1,21)<1.0, n.s.) was reported for subtracted changes in deoxyHb levels.

### Comparison between scanning strategy and hemodynamics

Multiple linear regression models for changes in oxyHb levels were not significant, as follows: L-IFG (self-face condition): *F*(3,18)<1.0, *p* = 0.93; R-IFG (self-face), *F*(3,18)<1.0, *p* = 0.75; L-IFG (familiar face), *F*(3,18)<1.0, *p* = 0.52; R-IFG (familiar face), *F*(3,18)<1.0, *p* = 0.64; L-IFG (unfamiliar face), *F*(3,18) = 2.4, *p* = 0.10; and R-IFG (unfamiliar face), *F*(3, 18) = 1.6, *p* = 0.29.

**Figure 7:**
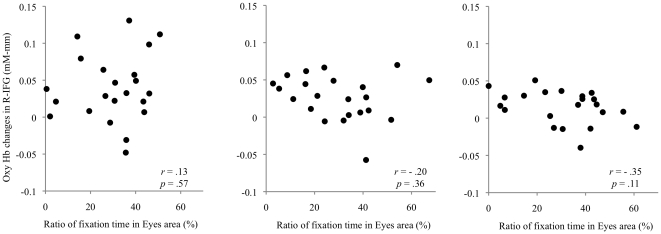
Relationships between proportions of fixation time in eye area and changes in oxyHb levels in the R-IFG (R-IFG: right for R). Three scatter graphs show (A) the self-face condition, (B) the familiar-face condition, and (C) the unfamiliar-face condition). None of the correlations reach significance at *p*<0.05.

There were no significant correlations between the ratios of fixation time for the eyes and hemodynamic changes of oxyHb levels in each nROI for all conditions (Fig. 7): L-IFG (self-face condition), *r* = 0.06, *p* = 0.80; R-IFG (self-face), *r* = 0.13, *p* = 0.57; L-IFG (familiar face), *r* = −0.32, *p* = 0.15; R-IFG (familiar face), *r* = −0.20, *p* = 0.36; L-IFG (unfamiliar face), *r* = −0.33, *p* = 0.14; R-IFG (unfamiliar face), and *r* = −0.35, *p* = 0.11.

## Discussion

To identify a face, we sample information by scanning several facial features, and then apply structural analysis and semantic encoding in neural networks. A previous study suggested that the scanning strategy was linked to brain activity, that is much time of fixation on eyes area intensified activities either in right amygdala or anterior fusiform gyrus [20]. However, our study raised a question about the positive relationship between scanning strategy and brain activities because the strategy was not even remotely related to the activities in frontal regions involved in face identification [18], [19].

In this study, scanning strategy did not differ significantly among the facial image conditions, which suggested that subjects scanned the images with analogous strategies irrespective of the kind of face. In contrast, hemodynamic activities around R-IFG changed among the conditions with increased activities in self-face condition as compared with those in familiar face condition. These implied that facial information was sampled in similar ways and then was processed in different ways. To illustrate this, we showed that there were neither multilinear connections nor correlations between fixation time on the eyes and changes in oxyHb levels in the bilateral IFG. On the other hand, previous studies showed that scanning strategy modulated brain activities in the fusiform gyrus, which played an important role in the structural analysis of facial images [20]–[22]. Thus, the fusiform gyrus might have been equally activated among the facial conditions because the face scanning strategy showed no difference among conditions in this study. Given the results of the present and previous studies, it is hypothesized that the IFG is activated via a different neural pathway from scanning strategy and activities in the fusiform gyrus. A few patient studies support such an interpretation [31]–[33]. They found that activities in the frontotemporal areas, including the IFG, might be driven by an atypical scanning strategy for face identification, independently or in parallel.

Present NIRS data showed that self-face recognition activated bilateral IFG. Moreover, the hemodynamic difference between self- and familiar face conditions was remarkable in R- IFG, not in L-IFG. Since the NIRS data were cancelled out the effect of “face recognition” itself by using that in unfamiliar face condition as a baseline, the equivalence between conditions in left hemisphere might be reflected on familiarity [34], that is, both of self- and familiar face images are “familiar” to the subjects. On the other hand, the R-IFG was significantly activated for self-face compared with familiar face. This might be caused by less reactivity to familiar face in R-IFG. Another possibility is that this area has a specificity to self-face processing. If the R-IFG also involved in only familiarity same as L-IFG, the difference between conditions would not show to be statistically significant. The difference was significant in only right hemisphere, which might imply that R-IFG was relevant not only with familiarity but also self-face effect as opposed to L-IFG.

Although these results do not directly confirmed the right dominancy of self-face recognition, the self-processing specificity in R-IFG could not be ruled out yet. Previous studies indicated the unique response in right side in behavioral [28], neurophysiological [35], and neuroimaging [19] studies, though a few suggested characteristics in left side [16], [36]. The present finding might reflect that self-face recognition and self-other discrimination are processed in a neural network including R-IFG [17]–[19]. Moreover, R-IFG is also a part of the cognitive system involved in processing self-information such as self-evaluation or self-relevance, and it is selectively activated in autobiographical memory retrieval [37], the process of comparing self-traits with others [38], [39], and judgments of self-face appearance [40]. In our task, an initial self-image was changed to that of another person in the morphing movie, which demanded that each subject should keep the first image in mind as a target for comparison in order to evaluate the gradually distorted face. Therefore, in the self-face condition, the subject performed self-evaluation during the task with some participants making casual remarks with regards to their own face being distorted, such as having an “odd feeling” and it being a “strange experience”. Activities in the R-IFG would have been relevant to both self–other facial discrimination and self-evaluation, mechanically or automatically.

On the other hand, the identity of a person in face recognition was not influenced by eye-movement variations as much as by hemodynamic changes. Contrary to a previous finding [10], subjects usually performed similar scanning strategy in all task conditions, and were more likely to fixate on the eyes and nose rather than on the mouth. This inconsistency might have resulted from different task demands. Another study [41] used three different tasks concerning the effects of facial familiarity on face processing, using eye-movement measures. The results indicated that, in specific tasks which focused on higher cognitive systems such as the recognition or identification of faces, the attention space of subjects tended to decrease and limited information from the attention space was processed. Our tasks, using morphing movies rather than static images [10], required the subject to evaluate the movie at every moment during the task because the image changed every 100 ms, so that they had to pay attention to limited areas, such as the eyes or nose, and the scanning strategy was similar among the facial conditions. Another possible explanation of the similar strategy is a cultural effect. Recently, a research group showed that East Asian people, such as Japanese and Chinese, made more fixations on the nose area of the face, and not the eyes or mouth, compared with Western Caucasian people during a facial recognition task [42]. The subjects in this study were all native Japanese, and thus most of them might have used a strategy that “would be optimal and economical to integrate information holistically” [42] by focusing on the center of the face, that is, the nose area. This area might be an appropriate spatial position when subjects scan the image globally, especially when watching a morphing movie, in which all facial features dynamically change at the same time. As a result of cultural effects and task traits, it is possible that the subjects performed an analogous strategy in all conditions.

Our task setting contained stopping the morphing movie and changing quickly into a static noise image when the subject responded. This might lead to a concern that hemodynamic changes simply reflect the time taken to evaluate stimuli. However, our results are still validated because RT as a behavioral index was not significantly different among the conditions in this study. RT was approximately 14 s, which means that the facial image contained about 70% of the target facial image when the subject responded. In a previous study there were no differences in RT between the self-face and the familiar face as the initial image [28]. Our results are similar to the findings of Keenan et al. [28] in the view of RT variation between the conditions. However, the subjects in both studies showed different RTs. In spite of having the same presentation time, 20 s, the RT differed between our results and those of Keenan et al. [28], where the subjects responded 9–10 s after the initial image appeared. Other studies using static morphed images showed that few subjects identified the facial image as a self-face when the image contained 60% of other facial factors [17]. These inconsistencies might be related to the presentation time of single frames. In previous studies, a single morphed frame was presented for 1000 ms [28] or a static image was presented for 4000 ms [17] rather than the short duration used in this study. If a single frame is presented for a long period with a large gap separating it from the previous frame, the subject can carefully observe each frame and then discriminate with a shorter RT. Our morphing movies were presented smoothly and maintained the subject's attention to the stimulus (more than half of the total stimulation time); hence, this task setting might be efficient for measuring gradual increases in hemodynamic activity in face identification tasks.

In conclusion, it is suggested that different facial images are sampled in similar ways but may be processed in ways different to those in face identification. Further studies are needed to identify the pathways whereby specific brain activities arise when similar scanning strategies are used, irrespective of the kind of facial image, because several regions other than frontal regions such as the IFG might play crucial roles in face identification. The combination of eye-tracking and hemodynamic activity measurement should throw light on this topic and allow systematic interpretation of face identification.

## References

[1] Bruce V (1982). Changing faces: visual and non-visual coding processes in face recognition.. Br J Psychol.

[2] Field TM, Cohen D, Robert G, Reena G (1984). Mother-stranger face discrimination by the newborn.. Infant Behav Dev.

[3] Bushnell IWR, Simion F, Butterworth G (1998). The origins of face perception.. The development of sensory, motor and cognitive capasities in early infancy: from perception to cognition.

[4] Amsterdam B (1972). Mirror self-image reactions before age two.. Dev Psychobiol.

[5] Tong F, Nakayama K (1999). Robust Representations for faces: Evidence from visual search.. J Exp Psychol Hum Percept Perform.

[6] Devue C, Van der Stigchel A, Bredart S, Theeuwes J (2009). You do not find your own face faster; you just look at it longer.. Cognition.

[7] Bredart S, Delchambre M, Laureys S (2006). One's own face is hard to ignore.. Q J Exp Psychol.

[8] Ellis H, Shepherd JW, Davies GM (1979). Identification of familiar and unfamiliar faces from internal and external features: some implications for theories of face recognition.. Perception.

[9] Young AW, Hay DC, McWeeny KH, Flude BM, Ellis AW (1985). Matching familiar and unfamiliar faces on internal and external features.. Perception.

[10] Althoff RR, Cohen NJ (1999). Eye-movement-based memory effect: a reprocessing effect in face perception.. J Exp Psychol Learn Mem Cog.

[11] Haith MN, Bergman T, Moore MJ (1977). Eye contact and face scanning in early infancy.. Science.

[12] Sadr J, Jarudi I, Sinha P (2003). The role of eyebrows in face recognition.. Perception.

[13] Northoff G, Heinzel A, de Greck M, Bermpohl F, Dobrowolny H (2006). Self-referential processing in our brain-a meta-analysis of imaging studies on the self.. NeuroImage.

[14] Platek SM, Wathne K, Tierney NG, Thomson JW (2008). Neural correlates of self-face recognition: an effect location meta-analysis.. Brain res.

[15] Sugiura M, Sassa Y, Jeong H, Miura N, Akitsuki Y (2006). Multiple brain networks for visual self-recognition with different sensitivity for motion and body part.. NeuroImage.

[16] Kircher TT, Senior C, Phillips ML, Benson PJ, Bullmore ET (2000). Towards a functional neuroanatomy of self processing: effect of faces and words.. Brain Res Cogn Brain Res.

[17] Uddin LQ, Kaplan JT, Molnar-Szakacs I, Zaidel E, Iacoboni M (2005). Self-face recognition activates a frontoparietal “mirror” network in the right hemisphere: an event-related fMRI study.. NeuroImage.

[18] Devue C, Collette F, Balteau E, Degueldre C, Luxen A (2007). Here I am: the cortical correlates of visual self-recognition.. Brain Res.

[19] Platek SM, Keenan JP, Gallup GG, Mohamed FB (2004). Where am I? The neurological correlates of self and other.. Brain Res Cogn Brain Res.

[20] Dalton KM, Nacewicz BM, Johnstone T, Schaefer HS, Gernsbacher MA (2005). Gaze fixation and the neural circuitry of face processing in autism.. Nat Neurosci.

[21] Morris JP, Pelphrey KA, McCarthy G (2006). Controlled scanpath variation alters fusiform face activation.. Soc Cogn Affect Neurosci.

[22] Morris JP, McCarthy G (2007). Guided saccades modulate object and face-specific activity in the fusiform gyrus. Hum.. Brain Mapp.

[23] Toyoda H, Kashikura K, Okada T, Nakashita S, Honda M (2008). Source of nonlinearity of the BOLD response revealed by simultaneous fMRI and NIRS.. NeuroImage.

[24] Leon-Carrion J, Damas-Lopez J, Martin-Rodriguez JF, Dominguez-Roldan JM, Murillo-Cabezas F (2008). The hemodynamics of cognitive control: the level of concentration of oxygenated hemoglobin in the superior prefrontal cortex varies as a function of performance in a modified stroop task.. Behav Brain Res.

[25] Marumo K, Takizawa R, Kawakubo Y, Onitsuka T, Kasai K (2009). Gender difference in right lateral prefrontal hemodynamic response while viewing fearful faces: a multi-channel near-infrared spectroscopy study.. Neurosci Res.

[26] Kawakubo Y, Kuwabara H, Watanabe K-i, Minowa M, Someya T (2009). Impaired Prefrontal Hemodynamic Maturation in Autism and Unaffected Siblings.. PLoS ONE.

[27] Matsuda G, Hiraki K (2006). Sustained decrease in oxygenated hemoglobin during video games in the dorsal prefrontal cortex: a NIRS study of children.. NeuroImage.

[28] Keenan JP, Freund S, Hamilton RH, Ganis G, Pascual-Leone A (2000). Hand response diffrences in a self-face identification task.. Neuropsychologia.

[29] Fukui Y, Ajichi Y, Okada E (2003). Monte Carlo prediction of near-infrared light propagation in realistic adult and neonatal head models.. Appl Opt.

[30] Van der Geest JN, Kemner C, Verbaten MN, Van Engeland H (2002). Gaze behavior of children with pervasive developmental disorder toward human faces: a fixation time study.. J Child Psychol Psychiatry.

[31] Bookheimer SY, Wang AT, Scott A, Sigman M, Dapretto M (2008). Frontal contributions to face processing differences in autism: evidence from fMRI of inverted face processing.. J Int Neuropsychol Soc.

[32] Grüter T, Grüter M, Carbon CC (2008). Neural and genetic foundations of face recognition and prosopagnosia.. J Neuropsychol.

[33] Kleinhans NM, Richards T, Sterling L, Stegbauer KC, Mahurin R (2008). Abnormal functional connectivity in autism spectrum disorders during face processing.. Brain.

[34] Druzgal TJ, D'Esposito M (2001). A neural network reflecting decisions about human faces.. Neuron.

[35] Gunji A, Inagaki M, Inoue Y, Takeshima Y, Kaga M (2009). Event-related potentials of self-face recognition in children with pervasive developmental disorders.. Brain Dev.

[36] Kircher TT, Senior C, Phillips ML, Rabe-Hesketh S, Benson PJ (2001). Recognizing one's own face.. Cognition.

[37] Vogeley K, Kurthen M, Falkai P, Maier W (1999). Essential functions of the human self model are implemented in the prefrontal cortex.. Conscious Cogn.

[38] Ochsner KN, Beer JS, Robertson ER, Cooper JC, Gabrieli JD (2005). The neural correlates of direct and reflected self-knowledge.. NeuroImage.

[39] Schmitz TW, Kawahara-Baccus TN, Johnson SC (2004). Metacognitive evaluation, self-relevance, and the right prefrontal cortex.. NeuroImage.

[40] Sadato N, Morita T, Itakura S (2008). The role of neuroimaging in developmental social psychology.. Brain Imaging and Behav.

[41] Stacey PC, Walker S, Underwood JDM (2005). Face processing and familiarity: evidence from eye-movement data.. Br J Psychol.

[42] Blais C, Jack RE, Scheepers C, Fiset D, Caldara R (2008). Culture shapes how we look at faces.. PLoS One.

